# Valvular Murmurs in Relation to the Treatment of Cardiac Disease

**Published:** 1908-03-07

**Authors:** R. W. Philip

**Affiliations:** Physician to the Royal Infirmary, Edinburgh.


### Hospital Clinics.

### VALVULAR MURMURS IN RELATION TO THE TREATMENT OF CARDIAC DISEASE.

By R. W. PHILIP, M.A., M.D., F.R.C.P.E., F.R.S.E., Physician to the Royal Infirmary, Edinburgh.

A Clinical Lecture delivered at the Royal Infirmary.

There are at present no fewer than twenty patients in my wards in the Royal Infirmary, who present cardiac murmurs in one or other variety. A certain number of the patients were admitted because of more or less serious heart disease, with associated cardiac murmurs. A good many, on the other hand, came to the wards because of some other illness, and the cardiac murmur was determined in course of examination. The presence of so many persons in this little community who present one or more physical signs of valvular disturbance has suggested to me, as the subject of to-day's clinical lecture, the significance of valvular murmurs in relation to treatment. It will be convenient and interesting if, in the foreground, I present to you a short analysis of the twenty cases to which I have just referred.

### Five Classes.

We shall divide these case according to their more conspicuous valvular manifestations into five classes, thus:—

I. *With mitral systolic murmur, eleven cases.*—These may be conveniently divided into three groups:—

(A) Three with systolic murmur, best heard in the mitral area, without other evidence of cardiac disease. The group includes one case of chlorosis and two of gastric ulcer.

(B) Six have a mitral systolic murmur, with less or more evidence of enlargement of the heart, but without any symptoms referable to the valvular lesion, as such. Indeed, the valvular lesion does not seem to have ever caused special trouble. The group includes one case of rheumatic fever, one of exophthalmic goitre, one of myxœdema, one of locomotor ataxia, and two of abdominal tuberculosis. Three of the patients are married, and have considerable families.

(C) Two patients were admitted to the ward with mitral incompetence and insufficient compensation. One is a case of acute heart failure, with grave dilatation, cardiac asthma, and pronounced cyanosis. The second has more chronic disturbance, with present failure of compensation, dyspnœa, dropsy, diminution of urine, etc.

II. *With mitral presystolic murmur, three cases.*—Of these, Group A contains one case, admitted into the ward on account of chorea. There have been no symptoms directly referable to the heart. Group B includes two cases, both of which present, along with the physical sign of mitral stenosis, evidence of more or less cardiac failure.

III. *With mitral systolic and presystolic murmurs, one case.*—Here, along with the physical signs of the valvular lesions, there is much cardiac failure, palpitation, dyspnœa, cough, dropsy, and deficient urine.

IV. *With aortic diastolic murmur, and less or more systolic roughening, four cases.*—Of these, Group A includes two patients, admitted to the ward on account respectively of sub-acute rheumatism, where the only acute symptom has been slight breathlessness on exertion, and of cerebral embolism, without other manifestation referable to the heart. Group B contains two patients, admitted to the ward with definite cardiac failure, including interference with forward flow (cerebral anæmia), pain, dyspnœa, dropsy, and diminished urine.

V. *With aortic systolic murmur alone, one case.*—The patient is an elderly man, admitted because of acute ptomaine poisoning. The arteries generally are very rigid and highly atheromatous. Notwithstanding this, he has had no cardiac symptoms whatever.

### Unsuspected Murmurs.

This rapid review of the twenty cases serves to demonstrate the fact that valvular disease frequently exists without causing the individual any inconvenience, and frequently without his knowledge. The experience of the ward corresponds with the everyday experience of the physician who has to do with insurance examination and other examinations of physical fitness for various public services. It is not at all uncommon for the candidate under examination to be apprised for the first time, as the result of that examination, that he has one or other valvular murmur. The presence of such murmur determines in no sense the question of treatment. It merely serves to raise the question, Does the case require treatment or not?

A large proportion of cases do not require treatment at all. In this sense the valvular murmur is analogous to a scar. It is a remnant, a historical mark, which may or may not have present significance. It cannot be too emphatically said that the detection of a murmur affords no warrant for the institution of drug treatment. Unhappily, it is all too frequent for the practitioner who makes the discovery to sit down at once and prescribe digitalis, strophanthus, or the like, which, far from helping the patient, may actually do him harm. If, following the lesion of which the murmur is but the outward sign, physiological equilibrium has been re-established, active medical treatment can do no good, and may conceivably upset the fine adjustment which Nature has achieved.

### Considerations Prior to Treatment.

In presence of a valvular murmur, then, how are we to determine whether the patient requires treatment? The answer is to be found in the answers to the following questions:—

(*a*) Are there any symptoms referable to the heart?

(*b*) What is the state of the heart muscle itself?
(*c*) What is the state of the vessels?
(*d*) Is there indication of circulatory disturbance in more distant organs?

In addition to these major considerations it is prudent, in passing judgment, to have regard to the following points:—

1. *The particular valve affected.*—The significance of valvular disturbance varies considerably with the valve affected. Thus, there are immediate risks in relation to aortic disease which are not present in relation to mitral disease. In the former case, even when compensation is sufficient, there remains the risk of sudden failure after extra effort or excitement. This must be thought of in the preventive rules which we prescribe to the patient. Further, aortic disease is, in a large proportion of cases, associated with degenerative processes in the aorta and coronary vessels. This may mean grave interference with nutrition of the heart, and all the accidents which this may entail.

Mitral valvular lesion affords a better outlook. I have, for example, just lost a patient, an old gentleman of 85, who, to my knowledge, had pronounced mitral incompetence for at least twenty-five years, and, judging by the history, probably for twice that period. Yet during the twenty-five years that I had the opportunity of observing him, there were hardly any heart symptoms until the very end.

2. *The age of the patient.*—Valvular lesion is more trying to children. This is perhaps due to the natural restlessness of the child and ignorance as to the meaning of symptoms and signs. Compensatory hypertrophy is readily achieved, but no less readily disturbed. The limits of compensation are speedily passed, and dilatation in excess supervenes.

3. *The sex and condition of the patient.*—Other things being equal, the male patient gives ground for greater anxiety. This is doubtless in chief part due to his more anxious and strenuous life, and the greater difficulty which commonly exists in respect of sufficiently long rest after acute illnesses. In part it is due to the more general indulgence in artificial stimulants of various kinds. To what extent the balance in favour of women may be disturbed, when the restless activity of the sex obtains completer satisfaction, remains to be seen. The extra risks which pregnancy and parturition entail are already well recognised.

4. *The patient's bill of health and habits.*—A rheumatic personal or family history must, of course, be taken into account. Short of actual recurrence of rheumatic fever, the heart is liable to suffer from time to time in slighter fashion. The same remark applies to influenza. The frequency with which influenza recurs in the same individual is remarkable, and the influenza toxin has a specially disturbing action on the heart. The kind of life which the patient leads, either of necessity or by choice, in respect of late hours, amount of sleep, regularity of meals, the use of alcohol, tobacco, etc., is most significant.

5. In all cases *the record of the patient's cardiac history* during the past three to five years is most helpful.

### General Principles of Treatment.

Keeping such considerations in view, we are in a position to turn again to the question of treatment. In view of the great variety of clinical type, we may lay down two general propositions:—

I. *If compensation be sufficient, endeavour to maintain it.*

Although a valvular murmur be present, the patient is in a condition of physiological equilibrium —an equilibrium which may be readily disturbed by unwise or hasty interference. The rules for the patient's guidance are simple and natural. They include: (*a*) The maintenance of a restful, easy life, regularity of hours, sufficiency of rest and sleep, the avoidance of excitement, rush, and worry. (*b*) Attention to the gastro-intestinal tract. The dietary should be simple, easily digested, and nutritious. Flatulent distension must be avoided, and movement of the bowels should be as regular as possible, and straining at stool avoided. (*c*) Gentle regulated exercise is desirable—*e.g.*, walking, gentle climbing, while more urgent physical strain or hurry is to be avoided. (*d*) Toxic influences should be excluded as much as possible. The amount of tobacco, alcohol, and other powerful stimulants should be minimised. Rheumatism, influenza, and other similar disorders must be carefully guarded against.

II. *If compensation be insufficient, endeavour to establish it.*

The symptomatic indications of failing compensation include irregularity of pulse and heart, dyspnœa, cough, hæmoptysis, dropsy, diminished urine, cerebral anæmia, with tendency to giddiness, fainting, etc., associated with physical signs of dilatation of the heart, and congestion of lungs, liver, kidneys, and other organs.

### Treatment of Failing Compensation.

In failure of compensation the indications for treatment are as follows:—

(*a*) Ensure as complete rest as possible, both muscular and nervous. This implies rest in bed, or on a couch, in suitable position, during the graver manifestations. The position of the patient varies according to the nature of the valvular lesion. Thus in mitral disease, the patient is commonly more comfortable propped up in bed, or leaning against the end of a couch, or even in an easy chair with his lower limbs supported. To insist on the more strictly recumbent posture, as one sometimes sees done in such cases, is a great mistake. In aortic disease, on the other hand, the patient is commonly better with his head low, although from time to time this requires modification. In most instances the patient, at least the adult patient, is a good judge as to the best position. In all cases the best position should be explained to the nurse.

### Regulation of the Diet.

(*b*) Attend to stomach and bowels. The dietary should err on the side of lightness. The food should be simple, nutritious, and concentrated. Soups are, as a rule, to be avoided. Three meals a day are best, thus:—

*Breakfast.*—One small cup of weak tea or coffee, a small piece of fresh fish or meat, preferably grilled, without sauce, and toasted stale bread, thinly spread with butter. *Luncheon.*—A little fresh fish or meat or chicken, boiled or grilled, toasted stale bread or rusk, well boiled onion, or spinach, or celery. *Dinner.*—Fresh fish, preferably whiting, grilled or boiled, beef, mutton or chicken grilled or roasted, toasted stale bread. Sometimes it is serviceable to give the larger meal in the middle of the day, rather than at night—the patient feels more comfortable and sleeps better. In addition to the above, a small cup of weak tea may be allowed in the afternoon, with a rusk or other dry biscuit.

The greatest care should be taken to exclude flatulence. This is in large part effected by simplicity and concentration in the dietary, as already indicated, and by the rigid exclusion of extras which may have flatulent tendencies. These are commonly known by the patient himself, though not necessarily avoided by him. It is a good rule to allow the patient a small glass of hot water to be taken slowly in the morning half an hour before breakfast and similarly the last thing at night. Where the patient has been accustomed to alcohol, a regulated amount may be allowed with one or both of the two chief meals, say, the equivalent of half an ounce of alcohol in a small quantity of water.

A daily movement of the bowels should be attained without effort to the patient. Where necessary, this should be determined by the exhibition of aloin, say ¼ grain, or a corresponding dose of cascara at lunch or dinner time. Nothing tends so much towards flatulent distension as the constipated habit. Where, in spite of such measures, flatulence continues, it is often relieved by an occasional dose of hydrarg. c. creta, say ½ to 1 grain, at dinner time. It is not necessary to give large doses. This may be combined suitably with an alkali—*e.g.*, bi-carbonate of sodium, a little rhubarb, and a simple carminative. If flatulent distension resist these simple measures, it is good practice to have recourse to rectal feeding by means of nutrient enemata and suppositories for several days.

### The Use of Drugs.

(*c*) Assist the circulation. This will be effected in two ways: (1) By the use of cardiac tonics, (2) in presence of dropsy, by depletion.

1. *Cardiac tonics* include especially digitalis and strophanthus. By digitalis we expect to give tone to the heart muscle, and so limit the tendency to dilatation. The cardiac action is slowed and the force and value of each contraction is increased. The peripheral arteries are contracted, and so the forward flow through capillaries and veins is hastened. Of favourable clinical effects when digitalis is proving serviceable, we note especially the slow pulse, increased arterial pressure, increased regularity of the heart, increase of urine, diminution of dropsy, and relief of more notable symptoms—*e.g.*, dyspnœa, cough, etc.

As to dosage, the two most generally serviceable preparations are (*a*) the tincture of digitalis, exhibited in ordinary cases, in doses say of 10 minims. In more pronounced cases of failing compensation, this dose must be considerably increased. The good effects of the drug may not be attained until 20 to 30 minims have been prescribed thrice daily. (*b*) Infusion of digitalis is a good preparation, which some patients tolerate better than the tincture. It may be exhibited in one or two tablespoonful doses. When a solid preparation is desirable, digitalis may be exhibited in powder, ½ to 2 grains, or Nativelle's granules, 1 to 3. Care will be taken, of course, to avoid cumulative toxic action of digitalis, which may show itself by increased rate and irregularity following on the preliminary slowing of the pulse. Digitalis, while specially serviceable in cases of mitral disease, is available in all cases, including aortic disease, with tendency to dilatation of the heart. It is particularly serviceable in aortic disease with secondary mitral incompetence.

In the same category as digitalis stands strophanthus, which has the same kind of action, save that it acts less definitely on the arterial wall. Hence it is more available in cases where the blood pressure is high. If digitalis be used in such cases, it is good practice to combine it with iodide of potassium, say 5 to 10 grains per dose. The dose of strophanthus is 5 to 20 minims of the tincture. Strophanthus has the disadvantage in some persons of readily inducing nausea, actual sickness, and diarrhœa.

Other drugs serviceable in the same direction are caffeine, which may be exhibited alone or in combination with digitalis, in doses of 5 to 10 grains of the citrate; and strychnine, which is most valuable and has special significance in cases where a breakdown in compensation is threatened, though it may be used in more serious conditions as well. It often saves from the graver condition by anticipation. It may be exhibited conveniently in form of the liquor, in doses of from 3 to 5 minims. Arsenic is serviceable, more particularly, in my experience, in the case of young subjects, when failure of compensation is threatened. Speaking generally, it is fully more valuable in aortic lesions, in which cases, having regard to the sclerosed or atheromatous condition of the vessels, it is conveniently combined, in the form of the liq. arsenicalis (say 3 to 4 minims) with iodide of potassium (say 5 grains). In other cases, where the blood pressure is low, it is suitably combined, as the liq. arsenici hydrochloricus (say 2 to 4 minims) with the liq. strychnini hydrochloridi. Lastly, where the Hb value of the blood is low, iron is indicated. This may be combined with arsenic, or strychnine, or both.

### Methods of Depletion.

2. *Depletion* may be effected by way of the kidneys. The action of digitalis as a diuretic is most potent. It may be assisted by other diuretics, such as acetate or citrate of potassium (30 grains) or diuretin (20 grains). The diuretic action of mercury must not be forgotten. The old-fashioned pill, consisting of powdered digitalis, blue pill, and squills, aa one grain, is most valuable in this connection. Depletion may be further attained by way of the bowel. The action of mercury in this connection is valuable. The use of concentrated salines, say sulphate of magnesium, in ½-oz. doses, or a combination of sulphate of magnesium and sulphate of sodium is most effective. Other hydragogue cathartics will suggest themselves. Where there is marked venous engorgement, depletion may be more quickly effected by means of venesection, or by wet cupping.

Treatment of Special Symptoms.

(*d*) Meet special indications as symptoms occur.

1. *Excessive dyspnœa* is largely met by position. In the case of mitral disease the patient should be propped up in bed, or if he feels more comfortable, should be allowed to sit up in an easy chair, suitably bolstered with pillows, and with his legs supported. Great care should be taken that the air of his room is both fresh and cool. If attention were more generally paid to this there would be less frequent call for the exhibition of oxygen.

Cardiac tonics will be pushed. This is one of the occasions when the more heroic doses of digitalis or of strophanthus may be necessary. In presence of effusions into the closed sacs, it is good practice to remove them. My belief is that it is wisest, where several sacs are involved, to tap one or other, preferably the left pleural sac first. It is extraordinary how quickly great dyspnœa yields when 10 or 20 oz. even are removed from a pleura.

For the subjective feeling of discomfort created by the dyspnœa there is no drug so valuable as morphine, which is most conveniently administered subcutaneously in small doses. Some relief may also be obtained by the aromatic spirit of ammonia, in drachm doses.

2. *Cough*.—Here the same sort of remedies are serviceable. Digitalis may suitably be combined with squills and spirit of chloroform. In conditions where the blood-pressure is high, doses of ipecacuanha are serviceable. Where the cough is frequent and irritating and interferes with sleep a teaspoonful of glycerin of codeine, 4 grains to the ounce, is of service.

3. *Hæmoptysis*.—This seldom calls for special treatment. Indeed, in many cases hæmoptysis is to be interpreted as the expression of a safety-valve mechanism, whereby the local congestion of the lungs is lessened. As to later manifestations of pulmonary thrombosis, it calls for the continued use of digitalis or other cardiac tonic.

4. *Palpitation and Pain*.—Palpitation tends to lessen under the use of cardiac tonics. For the thumping heart of hypertrophy, with little dilatation, minute doses of aconite may prove serviceable. Or relief may be had from the local application of ice over the præcordia. Pain is often dependent on gastro-intestinal disorder, more especially flatulent distension, and this must be attended to. Where the vessels are contracted, and the blood-pressure tends to be high, immediate benefit is commonly observed by the use of nitro-glycerin, grains $\frac{1}{100}$-$\frac{1}{50}$, or of the liquor trinitrini 1-2 minims, or in acute cases by inhalation of amyl nitrite. In more chronic cases the continued use of iodide of potassium is serviceable. In a considerable number of cases of cardiac pain, opium in some form will be found a useful addition to other treatment.

5. *Dropsy*.—The presence of dropsy is a clear indication for the maintenance and pushing of cardiac tonics. If digitalis and strophanthus seem to act insufficiently, their value may be increased by the addition of caffeine, say, 5 to 10 grains of the citrate, or of 20 grains of diuretin. Similarly depletion by way of the bowel tends to drain the fluid from the subcutaneous tissue. This may be effected by concentrated salines or by compound jalap powder. If fluid be present in the closed sacs, one or other of these should be tapped, preferably the pleura, if available. Where, in spite of everything, the fluid continues to collect in the subcutaneous tissue of the lower limbs and adjacent parts, slow continued drainage by means of Southey's capillary tube may be helpful.

6. *Gastro-intestinal disturbance*, more especially flatulence, is avoided by careful regulation of the dietary, and by the use of carminatives from time to time. Some of the well-recognised liqueurs are convenient and pleasant for the purpose, as, for example, Kümmel, Curaçoa, and Crème de Menthe. Of drugs for the purpose, perhaps the most valuable is the compound tincture of ether—sometimes known as Hoffman's anodyne—20-60 minims, or the tinctura chloroformi et morphinæ composita (minims 5-15). I have already referred to the significance of regular evacuation of the bowel, and the advantage sometimes to be derived from rectal feeding.

7. *Restlessness and Sleeplessness*.—In the first place, make sure that this is not due to gastro-intestinal disorder, either in the shape of overloading or flatulent distension. See that the patient's room is both fresh and cool, especially by night. It is often helpful to give a small cup of thin warm soup, or malted milk, at bedtime. With some patients a small tumbler of toddy, or a glass of champagne, is more effective. Of drugs, in minor cases the compound tincture of chloroform and morphine, which I have already referred to, or the compound tincture of camphor, may prove sufficient. When severe pain is present, opium in full dose may be required. Of hypnotics, in the absence of pain, perhaps the most available is paraldehyde (1-3 drachms).

Methodised Systems of Treatment.

This sketch of the treatment of valvular disease in its various manifestations would be incomplete did I not refer, in conclusion, to two methodised systems of treatment which have proved of service, more particularly in relation to chronic disease, when the heart-muscle is not too much prejudiced. These two systems are respectively Oertel's and the Nauheim treatment. Oertel's consists essentially, in first the careful limitation of the dietary, both in quantity and quality, and its exhibition in concentrated form with the absence of much fluid at meal times; and, second, the use of graduated exercise in walking and gentle hill-climbing, such as I have already proposed. The Nauheim treatment consists in, first, the use of mineral baths, rich in sodium chloride, and containing more or less of calcium chloride and carbonic-acid gas; and, second, so-called "resistance exercises," practised so as to involve for the patient a minimum of effort and no fatigue. Into the details of these it is impossible to enter further on the present occasion.

